# Genes and Mechanisms Involved in the Generation and Amplification of Basal Radial Glial Cells

**DOI:** 10.3389/fncel.2019.00381

**Published:** 2019-08-20

**Authors:** Maxime Penisson, Julia Ladewig, Richard Belvindrah, Fiona Francis

**Affiliations:** ^1^Inserm, Institut du Fer à Moulin, Sorbonne Université, Paris, France; ^2^Inserm UMR-S 1270, Paris, France; ^3^Institut du Fer à Moulin, Paris, France; ^4^Central Institute of Mental Health, Medical Faculty Mannheim, Heidelberg University, Mannheim, Germany; ^5^Hector Institute for Translational Brain Research (gGmbH), Mannheim, Germany; ^6^German Cancer Research Center, Heidelberg, Germany

**Keywords:** cortical development, neural progenitor cells, basal radial glia, cell division, spindle orientation, adhesion, signaling pathways

## Abstract

The development of the cerebral cortex relies on different types of progenitor cell. Among them, the recently described basal radial glial cell (bRG) is suggested to be of critical importance for the development of the brain in gyrencephalic species. These cells are highly numerous in primate and ferret brains, compared to lissencephalic species such as the mouse in which they are few in number. Their somata are located in basal subventricular zones in gyrencephalic brains and they generally possess a basal process extending to the pial surface. They sometimes also have an apical process directed toward the ventricular surface, similar to apical radial glial cells (aRGs) from which they are derived, and whose somata are found more apically in the ventricular zone. bRGs share similarities with aRGs in terms of gene expression (*SOX2*, *PAX6*, and *NESTIN*), whilst also expressing a range of more specific genes (such as *HOPX*). In primate brains, bRGs can divide multiple times, self-renewing and/or generating intermediate progenitors and neurons. They display a highly specific cytokinesis behavior termed mitotic somal translocation. We focus here on recently identified molecular mechanisms associated with the generation and amplification of bRGs, including bRG-like cells in the rodent. These include signaling pathways such as the FGF-MAPK cascade, SHH, PTEN/AKT, PDGF pathways, and proteins such as INSM, GPSM2, ASPM, TRNP1, ARHGAP11B, PAX6, and HIF1α. A number of these proteins were identified through transcriptome comparisons in human aRGs vs. bRGs, and validated by modifying their activities or expression levels in the mouse. This latter experiment often revealed enhanced bRG-like cell production, even in some cases generating folds (gyri) on the surface of the mouse cortex. We compare the features of the identified cells and methods used to characterize them in each model. These important data converge to indicate pathways essential for the production and expansion of bRGs, which may help us understand cortical development in health and disease.

## Introduction

The cerebral cortex is the seat of complex brain functions and is a highly organized and compartmentalized structure. Its development relies on several progenitor types. These cells have specific morphologies, somal localizations, and division characteristics. They produce neurons that migrate to the cortex, their axons and dendrites forming neural circuits that underlie brain functions. Glial cells are also produced from cortical progenitors, providing support to neurons and playing a role in brain homeostasis. Cortical malformations are characterized by intellectual disability and/or epilepsy, and are associated with abnormalities in cortical structure and/or the number of neurons (Desikan and Barkovich, 2016; Romero et al., 2018). They are caused by various defects in cortical development such as during cell proliferation and migration, and englobe different phenotypes such as microcephaly (reduction of cerebral volume), lissencephaly (cortical layer disorganization and impact on folds), or heterotopias (presence of gray matter within the white matter). It is important to understand different aspects of cortical development and processes occurring during evolution, in order to predict pathogenic mechanisms which occur in human cortical pathology.

At early stages of neurodevelopment and before the start of neurogenesis at approximately gestational week (GW) 5–6 in humans (Zecevic, 2004; Howard et al., 2008; Meyer et al., 2018) and embryonic day (E) 10.5 in the mouse (Dennis et al., 2016), the neural tube is composed of neuroepithelial cells (NEs) which form a single layered pseudostratified epithelium (Götz and Huttner, 2005). They are the primary progenitor cell type as all neurons and glial cells (apart from microglia) will descend from NEs. These cells display a particular behavior during the cell cycle, called interkinetic nuclear migration, during which nuclei migrate apically toward the ventricular surface to perform mitosis and then basally toward the pial side of the neuroepithelium, where they enter S-phase. This phenomenon gives rise to nuclei localized at different levels along the apical-basal axis depending on the cell cycle phase, which gives the neural tube its pseudostratified appearance. NEs express markers such as Nestin (intermediate filament protein), Sox2 (transcription factor), and Notch.

**FIGURE 1:**
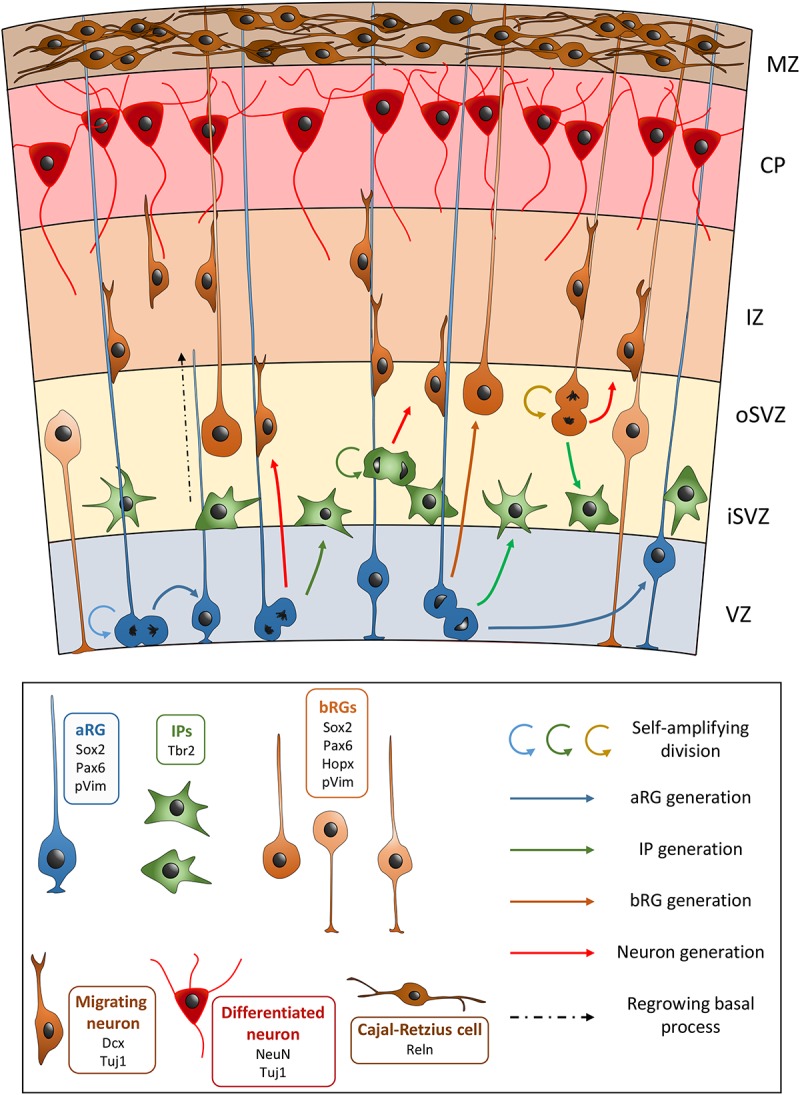
Schematic view of the development of the embryonic neocortex. VZ, ventricular zone; iSVZ, inner subventricular zone; oSVZ, outer subventricular zone; IZ, intermediate zone; CP, cortical plate; MZ, marginal zone. aRG, apical radial glial cell; bRGs, basal radial glial cells (different bRG morphotypes are shown); IP, intermediate progenitor. In the key, common genetic markers are cited with each cell type.

Neuroepithelial cells first perform symmetrical divisions in order to amplify the number of progenitors (Huttner and Kosodo, 2005). Upon the start of neurogenesis, NEs begin to perform asymmetrical divisions to produce neurons and other progenitors, as well as to self-renew. This production leads to the thickening of the tissue and the cortical wall becomes divided into several regions: the ventricular zone (VZ), the subventricular zone (SVZ), the intermediate zone (IZ), the sub-plate, the cortical plate (CP), and the marginal zone (MZ) (Figure 1). The production of neurons is also concomitant with the appearance of another type of progenitor cell derived from NEs, apical radial glia cells (aRGs) (Götz and Huttner, 2005; Borrell and Götz, 2014). aRGs are highly polarized, their cell bodies are localized in the VZ and they possess two processes: an apical process extending to the ventricular surface, and a long basal process, connecting to the pial surface. Like NEs, aRGs perform interkinetic nuclear migration, with nuclear movement restricted to the VZ: S phase occurs in the basal region of the VZ, while mitosis takes place at the ventricular surface. aRGs express markers such as Sox2, Pax6, Nestin, and Vimentin (Vim). In rodents, during early corticogenesis, most mitoses performed by aRGs are horizontal (i.e., cleavage planes perpendicular to the ventricular surface). These cells can self-renew through these symmetrical divisions, but they can also produce immature neurons, as well as basal progenitors (BPs). In the rodent, the most abundant BPs are termed intermediate progenitors (IPs), which express Tbr2. IPs are localized in the SVZ and in the rodent they generally divide once to produce two immature neurons (Borrell and Götz, 2014). In primate brains they are known to divide several times amplifying the pool of IPs, as well as generating neurons (Betizeau et al., 2013). It has been shown that aRGs can also produce another type of BP with characteristics of a radial glial cell, and these are termed outer or basal radial glial cells (bRGs) (Hansen et al., 2010; Borrell and Götz, 2014). These cells have been found to be enriched in the developing cortex of gyrencephalic species such as primates or the ferret, their somata are localized in the inner and outer SVZ (iSVZ and oSVZ, the latter a superficial sub-region representing the largest proliferative compartment in these species from mid-corticogenesis, see section “oSVZ Genesis, Cell Composition, and Function” for more information) (Hansen et al., 2010). The discovery of the neurogenic potentiality of bRGs is relatively recent (Hansen et al., 2010). bRGs were originally described as having a basal but often no apical process (Hansen et al., 2010), however, they can exist in different forms in primates (Betizeau et al., 2013). They are generated from aRGs after an asymmetrical oblique or vertical division (i.e., horizontal cleavage planes parallel to the ventricular surface). The daughter cell that is destined to become a bRG will receive the basal process, and the other cell can either become an immature neuron, an IP, or an aRG that remains attached to the ventricular surface and that will regrow its basal process (Shitamukai et al., 2011; LaMonica et al., 2013). Being highly neurogenic, bRGs are thought to be important for gyrencephalic brain development, especially associated with the apparition of cortical folds (gyri and sulci). These cells are few in number in lissencephalic species (such as rodents) (Wang et al., 2011; De Juan Romero and Borrell, 2015; Fernández et al., 2016; Borrell, 2018).

Cortical glutamatergic neurons are produced via direct or indirect neurogenesis, either from aRGs, or through IPs or bRGs, respectively (Malatesta et al., 2003). When they are born, they go through a multipolar phase, before adopting a bipolar morphology and migrating radially toward the CP, crossing the IZ. Here they settle in an inside-out fashion with early-born neurons destined for the deeper layers of the cortex, whilst later-generated neurons are destined for the more superficial layers (closer to the pia) (Sekine et al., 2014; Hatanaka et al., 2016). Cajal-Retzius cells reside in the MZ, and play important roles in the correct organization of the layers (Martínez-cerdeño and Noctor, 2014). Thus the layered structure of the cortex arises. Cortical neurons will send axons toward their targets and start connecting with neurons in other cortical and subcortical regions, forming the neural circuits underlying complex functions of the cortex.

## Characteristics of bRGs

### Identification of bRGs in Different Models

The importance of bRGs in cortical development has been highlighted in recent years by an increasing body of evidence accumulated in different models. We summarize here essential bRG data, contributed by different complementary approaches.

While many insights concerning bRGs have been gained through the use of human tissue, acquiring and performing analyses on human fetal brain tissue can be a potent limiting factor. Other gyrencephalic species, such as primates which are close to humans phylogenetically, or ferrets, have also been used as alternatives. Ferret bRGs are similar to human bRGs, they are abundant, possess a basal process, can proliferate and are Pax6+/Sox2+. The use of transgenic animals or performing electroporation in developing ferret brains (Kawasaki et al., 2012) can allow study of the involvement of certain genes potentially involved in bRG generation (described further in section “Molecular Mechanisms Associated With the Generation and Amplification of bRGs, Including bRG-Like Cells in the Rodent”).

Brains of a lissencephalic species, the mouse, have also been shown to have bRGs during cortical development (Wang et al., 2011). Using *in utero* injection at E12 to express GFP in progenitors, the team of Wang showed the presence of cells located in the upper part of the SVZ and possessing a basal process, but no apical process. Like human, cells expressed Pax6, Sox2, and phosphorylated Vimentin (pVim). However, these cells were few in number, as they only accounted for 5–10% of total between E12 and E18 (Wang et al., 2011). Furthermore, contrary to human and primate bRGs, none of the murine bRG-like cells were also found to express Tbr2. While being capable of self-amplifying divisions, as human cells, they were found to produce neurons but not IPs (Hansen et al., 2010; Wang et al., 2011). These results suggest that murine bRG-like cells are few in number and are functionally distinct from human bRGs. However, a recent study also showed that in the late developing mouse medial neocortex, abundant Hopx+ bRGs were present (Vaid et al., 2018). At E18, these cells could produce neurons and RNA sequencing showed that they resembled human bRGs transcriptionally. This population could hence serve as a good model to study bRGs. Furthermore, genetic manipulation to express or repress genes involved in bRG generation in human, has been performed in the mouse by various groups, and this can lead to an artificial bRG enrichment in the murine cortex (described further in sections “FGF-MAPK Pathway,” “Hif1α,” “SHH Signaling,” “Pax6,” “mSWI/SNF Subunits BAF170 and BAF155,” “INSM1,” “GPSM2 and Notch-Delta,” and “Human and Primate Evolutionary Inventions”).

### Gene Expression Profile

Since the identification of bRGs, there have been increasing transcriptome studies focused on comparisons of rodent and human cortex, to characterize the expanded oSVZ and bRGs. For example, Fietz et al. (2012) used laser capture microdissection to separate proliferative zones and the CP in mouse (E14.5) and human (13–16 GW) fetal neocortex. Differentially expressed genes were identified between the zones, including species-specific differences, highlighting the importance of the extracellular matrix on the proliferative and self-renewing properties of progenitors. With improved technologies, higher resolution methods took advantage of cellular heterogeneity and different cell abundancies in individual human fetal brain sections, identifying modules of co-expressed genes from brain section transcription profiles (Lui et al., 2014). Searching for genes specifically expressed in human bRGs (vs. mouse), 18 candidate genes were identified (including *PDGFD*, *BMP7*, and *FAM107A*). PDGFD-PDGFRb signaling was found to be important in human but not mouse cortex and involved in bRG production (see section “PDGFD Signaling”). Florio et al. (2015) also used fluorescent activated cell sorting (FACS) to characterize the transcriptomes of different human fetal cortex cell populations (Florio et al., 2015) which they compared to equivalent cells from mouse embryonic neocortex. Apical plasma membrane markers helped distinguish aRGs from bRGs. Human bRGs showed a different gene expression profile compared to mouse bRGs, and some genes were identified that were not expressed in mouse (e.g., human-specific gene *ARHGAP11B*, see section “ARHGAP11B”).

Gene expression was also characterized in the ferret, for example by performing transcriptomic analysis of microdissected proliferative layers from regions of prospective gyri and sulci at P1 (equivalent to mouse E15, De Juan Romero and Borrell, 2015). As well as identifying differentially expressed genes in the oSVZ, they found that gene expression levels changed abruptly and repeatedly across the cortex, distinguishing multiple domains. Martinez-Martinez et al. followed this up by studying earlier ferret stages (see section “Gene Expression Profile” and “Human and Primate Evolutionary Inventions”), searching for differential gene expression in the VZ between three key stages: E30, E34, and P1 (Martínez-Martínez et al., 2016). This led to the identification of key genes (e.g., downregulated *Trnp1* and *Cdh1*) important for the initial production of bRGs. 54 genes changed their expression between E30 and E34, and 1822 between E34 and P1.

Johnson et al. (2015) used fluorescent sorting approaches to profile cell populations of interest from dissociated human cortical tissue using the expression of surface markers to differentiate the cells. They identified transcription factors downstream of Neurogenin 2, enriched in BPs as compared to aRGs. Microfluidic single cell RNA sequencing techniques were also applied to these cells to identify enriched non-apical BP genes, including in ferret. Similar approaches were also used by Pollen et al. (2014, 2015) without previous enrichment of the cell populations. 67 candidate marker genes were identified that strongly correlated with the human BP/bRG population (Pollen et al., 2014, 2015). Genes were highlighted related to extracellular matrix formation, migration and stemness, including *TNC* (Garcion et al., 2004), *PTPRZ1* (Baldauf et al., 2015), *FAM107A* (Kiwerska et al., 2017), *HOPX* (Yap et al., 2016), and *LIFR* (Wu et al., 2018). Importantly, LIFR/STAT3 signaling was found to be required for bRG cell cycle progression and selectively expressed by bRGs (Pollen et al., 2015). These cells were hence found to express genes important for self-renewal pathways and stemness, not detected in aRGs (which receive signals from the ventricles), and to have the capacity for extensive proliferation, as also suggested by the fact that many of the genes have roles in various types of cancer.

Thus, several studies have focused on analyzing the transcriptome of bRGs in the human brain in order to better understand their specificity and how, when and why they are enriched in gyrencephalic brains (Stahl et al., 2013; Johnson et al., 2015; Pollen et al., 2015; Thomsen et al., 2015; Liu et al., 2017). While sharing many similarities with aRGs in terms of gene expression, with both cell types expressing genes such as *PAX6*, *Nestin*, *PDGFD*, *SOX2*, and *VIM*, bRGs express specific genes, which along with other characteristics, makes them a distinguishable progenitor cell type compared to aRGs.

### Morphology

Basal radial glial cells were originally described in human brain tissue to have a basal process extending to the pial surface, but no apical process, using pVim and DiI staining, in two different studies (Fietz et al., 2010; Hansen et al., 2010). DiI staining later revealed that cycling oSVZ progenitors possessed basal processes, and in some instances, also apical processes even though these did not reach the ventricular surface (Betizeau et al., 2013). The basal process also displayed varicosities (small bulges highlighted by Vim) that appear during M-phase. Later, it was shown that bRGs were highly heterogeneous in the macaque developing cortex: bRGs with a basal process, but no apical process, or with both basal and apical processes, and bRGs with only an apical process were described (Betizeau et al., 2013).

### bRG Division: Mitotic Somal Translocation

Just before cytokinesis of bRGs, a rapid somal translocation occurs along the basal fiber toward the pial surface. This key step is crucial to position subsequent cell production of bRGs and IPs in the iSVZ/oSVZ and thus bypass potential overpopulation of progenitors in the VZ (Hansen et al., 2010). This cellular process named mitotic somal translocation is tightly regulated as for interkinetic nuclear migration of NEs and aRGs, and therefore could potentially fulfill similar functions (Hansen et al., 2010) to regulate the production of progenitors. This process may even be at the origin of the radial expansion of the oSVZ (Lui et al., 2011). It has been suggested that molecular pathway similarities exist between these two mechanisms (LaMonica et al., 2012). However, these cellular processes present in fact striking differences: to pull the nucleus toward the apical surface, interkinetic nuclear migration requires microtubule interacting components such as dynein and Lis1 (Gambello et al., 2003; Tsai and Gleeson, 2005; Taverna and Huttner, 2010), while mitotic somal translocation seems to depend on non-muscle myosin II and the Rho effector ROCK. Moreover, this somal translocation occurs independently from centrosome movement (Ostrem et al., 2014).

## Generation, Amplification and Function of bRGs and Their Role in the Formation of Folds

### Cell Mechanisms Important for bRG Generation: Spindle Orientation and Cell Adhesion

Mitotic spindle orientation is highly regulated during aRG mitosis and well-characterized proteins involved in this process are GPSM2 (LGN), Dynein, NuMA, Dlg1, and Afadin (reviewed in di Pietro et al., 2016). Spindle orientation, along with the highly polarized nature of RGs, allows the segregation of soluble factors to each daughter cell, giving rise to new aRGs, IPs, bRGs or immature neurons. Indeed, when an aRG divides, the cleavage orientation may predict the fate of the daughter cells: bRG generation is positively correlated with oblique or horizontal cleavage angles (vertical divisions), the more basal cell migrating out of the VZ, while self-enriching divisions producing two aRGs are correlated with vertical cleavages planes (horizontal divisions) (Lancaster and Knoblich, 2012; LaMonica et al., 2013). LaMonica et al. (2013) performed time-lapse imaging in human brain slices at GW 18 to study the fate of cells after aRGs had performed division. Observing 70 divisions, 15 induced the generation of a bRG, characterized by basal fiber maintenance after division and cell translocation. All these bRGs were generated from horizontal (14) or oblique (1) cleavage angles, with the most vertical cell becoming the bRG (LaMonica et al., 2013). If the other daughter cell was destined to become an aRG, these cells were shown to regrow their basal processes in a Notch-dependent fashion, suggesting a self-renewal process to maintain the pool of APs (Shitamukai et al., 2011; LaMonica et al., 2013). These data are compared to previous results showing that in the mouse developing cortex, depending on the age, aRGs could carry out symmetric and asymmetric divisions while maintaining a near vertical spindle angle for both (Noctor et al., 2008). While being slightly slanted to one side due to one daughter cell inheriting the basal process, these horizontal divisions between E13 and E15 led to a majority of self-amplifying divisions, while between E16 and E19, the vast majority of dividing aRGs produced two different cells. This suggests that mitotic spindle orientation is not the sole mechanism required to adjust cell fate after division.

In the ferret it was shown in lineage experiments that oSVZ bRGs are predominately produced from aRGs during a restricted time period (Martínez-Martínez et al., 2016), when self-consuming divisions produced bRGs which seed the oSVZ. Amongst genes showing changed gene expression at E34 (e.g., compared to E30) was the downregulated adherens junction *Cdh*1 (*E-cadherin*) gene. Thus an influence on adherens junctions appears important for cell delamination. This is discussed in more detail in sections “mSWI/SNF Subunits BAF170 and BAF155,” “INSM1,” and “TBC1D3.”

### Cell Cycle Properties of bRGs

Features of bRG divisions are complex, and while they are akin to aRGs, some aspects in comparison with other progenitors make them unique. First, contrary to initial studies showing limited proliferation of bRGs (Hansen et al., 2010), it is now known that they can undergo not only two but several rounds of proliferation (Betizeau et al., 2013), like aRGs of the VZ. IPs perform divisions with random cleavage planes, unlike the vast majority of bRG divisions where the upper cell conserves the basal process and performs mitotic somal translocation (Hansen et al., 2010; LaMonica et al., 2012, 2013). Secondly, despite their delamination from the VZ, bRG amplification is strictly controlled. Unlike other well-structured epithelia, where the cohesiveness of cellular architecture strictly controls their proliferation, bRGs have other mechanisms, and despite their delamination, maintain a rigorous cell cycle regulation. Local extracellular matrix molecules secreted around the bRGs might create a microenvironment in the oSVZ in order to control the proliferation of β1-integrin+ bRGs (Fietz et al., 2010, 2012; Pollen et al., 2015), in conjunction with other signals received in the basal process. Their cell cycle length is also strikingly different from what is known for rodent progenitors. While the total duration of the progenitor cell cycle is increased according to developmental stages in rodent, in contrast, in primate cortex, the total duration of the cell cycle of bRGs is decreased (Haven, 1998). One of the direct consequences of this, is the acceleration of bRG proliferation within the oSVZ which correlates with the expansion of the cortex (Betizeau et al., 2013).

### oSVZ Genesis, Cell Composition, and Function

The SVZ has a very important role during brain development in gyrencephalic species, especially in primates. It becomes divided in two regions, the apical iSVZ, and the basal oSVZ between GW12 and 16 in humans (Hansen et al., 2010; Lewitus et al., 2013). Two major progenitor cell types can be found primarily in each of these regions. Cells enriched in the iSVZ mainly resemble IP cells present in the rodent SVZ and are TBR2+ while progenitors enriched in the oSVZ are mainly RG-like cells. In the human developing cortex, TBR2 staining is mostly present in the iSVZ, with the vast majority of cells expressing both TBR2 and PAX6. At GW16, 90% of progenitors in the iSVZ express TBR2 and 50% of progenitors express PAX6, while less than 5% are TBR2+ and more than 90% are PAX6+ in the oSVZ (Fietz et al., 2010). Out of the PAX6+ cells, 90% are SOX2+ and many express KI67, hence bRG-like cells present in the oSVZ are dividing progenitors. While neurogenesis from aRGs and IPs is important, oSVZ cells represent a large proportion of the proliferative cells throughout cortical development in humans (Hansen et al., 2010), suggesting the importance of bRGs during corticogenesis.

In the macaque, corticogenesis starts at E45. Different staining methods have shown the correlation between a wave of corticogenesis and the appearance of the oSVZ that occurs between E55 and E65 in the monkey and expands up to E94 (Smart et al., 2002; Betizeau et al., 2013). For comparison, in the ferret oSVZ seeding and production of upper-layer neurons starts at around E33 and the oSVZ remains up until P1 (with birth at E42) (Fietz et al., 2010; Martínez-Cerdeño et al., 2012). In the macaque iSVZ, the vast majority of cells express TBR2 (60–80% of TBR2+/PAX6+ and 5–30% TBR2+ only) with less than 15% of PAX6+ only cells. In the oSVZ, it is the opposite with the majority of cells expressing PAX6+ (25–50% of TBR2+/PAX6+ cells and 20 to 35% expressing PAX6+ alone), with only 10–20% of TBR2+ cells, which is similar to human development (Betizeau et al., 2013). A high proportion of cells located in the oSVZ that are proliferative present the typical morphology of bRGs: 46% of cells are SOX2+/p-VIM+, and display a basal process. Different experiments using GFP-expressing adenovirus and co-staining for BrdU and EdU (thymidine analogs) showed the high proliferative capacities of these cells, capable of symmetric or asymmetric division. Initial studies showed that bRGs can not only expand the pool of bRGs themselves (with two rounds of division) but also generate TBR2+ neuronal precursor cells (LaMonica et al., 2012). In human, an increase in size of the oSVZ during GW13 to GW15.5 is proportional to the expansion of the proliferative population (Hansen et al., 2010). Detailed studies in the macaque revealed the presence of a more heterogeneous population of bRGs than initially expected (Betizeau et al., 2013) hence the proportion of bRGs could have been underestimated initially on the basis of morphology alone.

### Contribution of bRG Proliferation to the Formation of Gyri and Sulci

The origins of formation of gyri and sulci in primate brains are still highly debated (Borrell, 2018; Kroenke and Bayly, 2018). Up to mid-gestation, in humans, the cortex is relatively agyric. From GW25 (Bystron et al., 2008), the CP expands rapidly and the folding process begins. What are the causes at the basis of the apparition of folds? These are very likely multiple. First, in recent years, compelling evidence from genetic manipulation of genes and pathways (described in section “Molecular Mechanisms Associated With the Generation and Amplification of bRGs, Including bRG-Like Cells in the Rodent”) revealed an amplification of bRGs as well as the formation of folds (Stahl et al., 2013; Ju et al., 2016; Wang et al., 2016a; del Toro et al., 2017). In the ferret, the modulation of BP/bRG proliferation can regulate gyri formation (Reillo et al., 2011; Nonaka-Kinoshita et al., 2013; Matsumoto et al., 2017). Changes in bRG proliferation can induce drastic consequences in radial fiber organization and subsequently induce folds. Second, regulation of neuronal migration (for instance by modulation of Flrt adhesion molecule expression) could potentially induce neuronal clustering underlying fissure formation (del Toro et al., 2017). Also, a balance between outward and inward forces generated by the modulation of bRG proliferation and neuronal migration, respectively, contributes to the formation of folds by mainly changing the radial organization. Third, a tangential dispersion of neurons arising from bRGs, coupled with the maturation of the neurons positioned in the different cortical layers is also a major component of cortical folding. The increased complexity of dendritic arborization and axon development contributes to changes in the stiffness of the cortical structure and thus also promotes the apparition of forces generating the formation of gyri and sulci. These modifications in the biophysical properties of the neural tissue have been reproduced in polymer gel models or in human organoid culture systems and consistently generated artificial folding (Tallinen et al., 2014, 2016; Karzbrun et al., 2018). Fourth, recent studies involving extracellular matrix molecules identified a certain number around bRGs in the oSVZ (Fietz et al., 2010, 2012; Pollen et al., 2015). Some of these molecules such as Lumican, Collagen I and HAPLN1 (HLC) concentrate principally in these germinal zones, and when their expression is modulated by artificial treatment they have the ability to change the stiffness of the tissue contributing also to fold formation (Long et al., 2018; Wianny et al., 2018). Consequently, although forces that are generated at the end of neurogenesis can contribute to the gyrification process, bRGs by their morphology (with a long basal process), their specific basal localization, their proliferative capacity and their neuron production, are likely to be major players in the events initiating cortical folding.

## Molecular Mechanisms Associated With the Generation and Amplification of bRGs, Including bRG-Like Cells in the Rodent

**FIGURE 2:**
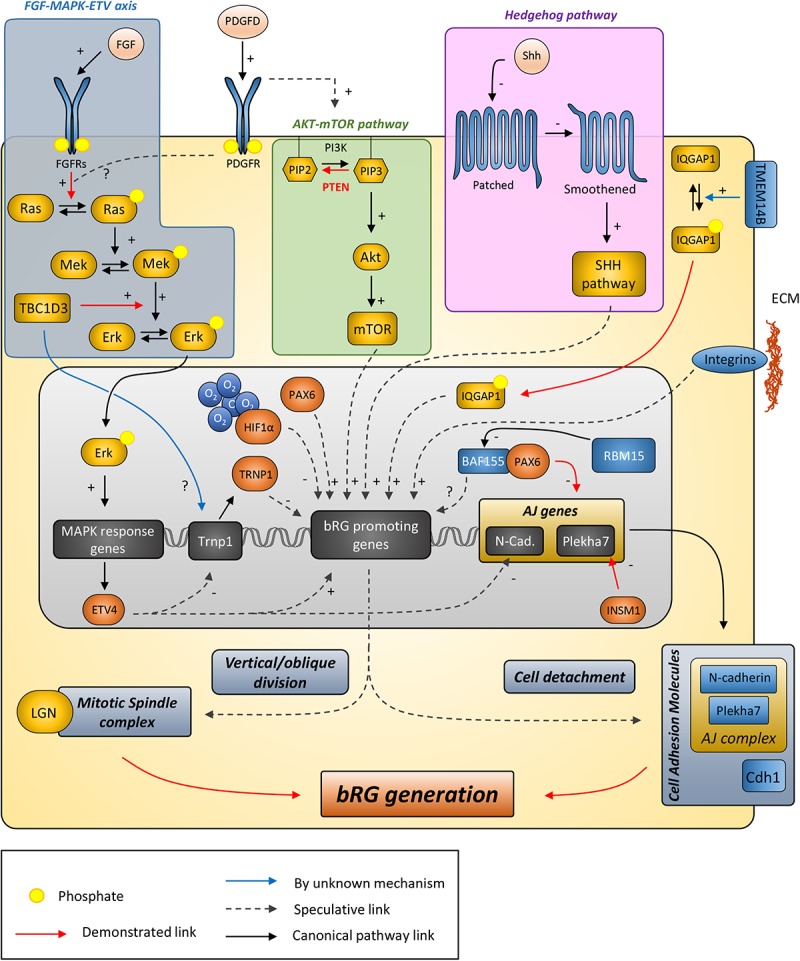
Putative model describing the signaling pathways and other genes suggested to be involved in bRG generation. Canonical pathways currently demonstrated to promote bRG production include the FGF-MAPK axis, PDGFD signaling, PTEN/AKT/mTOR, and SHH pathways. Human/hominoid specific genes depicted in this figure include TBC1D3, a RABGAP protein that promotes Erk signaling and appears to repress Trnp1 expression, and TMEM14B, a transmembrane protein that promotes IQGAP1 translocation to the nucleus to regulate cell cycle progression. ARHGAP11B and Aspm are not indicated here as their function are either unclear or they do not obviously fit in the pathways with current knowledge. AJ, adherens junction; ECM, extracellular matrix.

**FIGURE 3:**
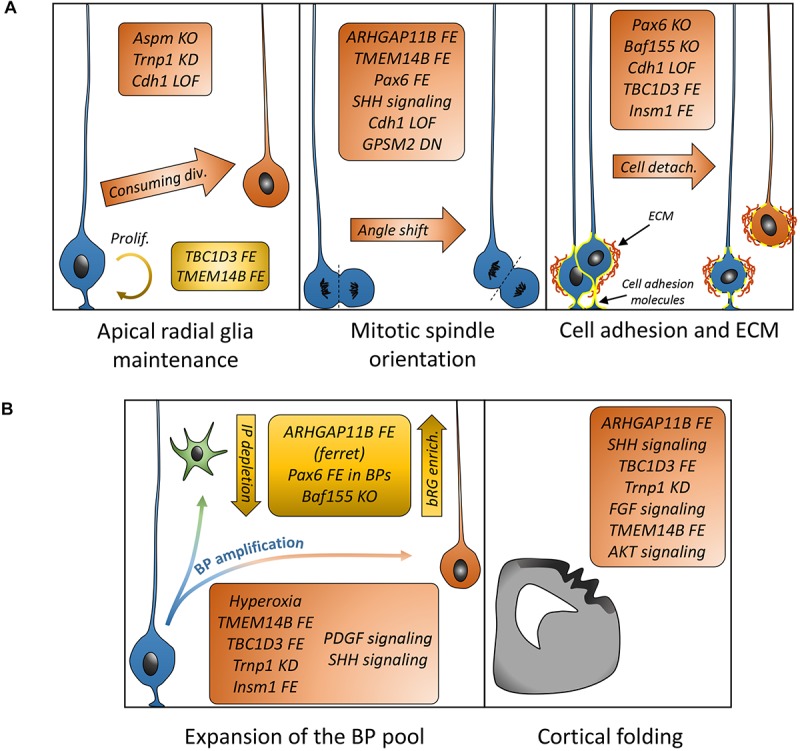
Mechanisms and genes associated with bRG generation/amplification. **(A)** List of genes demonstrated to be associated with cellular mechanisms (aRG maintenance or depletion, mitotic spindle orientation, and cell adhesion) leading to bRG generation. **(B)** List of genes or treatments leading to differential basal progenitor pool expansion and apparition of folds in the mouse (TBC1D3 FE, Trnp1 KD, and TMEM14B) or human organoids (AKT signaling) or modified folding in the ferret (ARHGAP11B FE, FGF signaling). FE, forced expression; KD, knockdown; LOF, loss of function; DN, dominant-negative.

Understanding how bRGs are generated has been a priority for many research groups since 2010. As well as human tissue, using the ferret, or artificially enriching bRG-like cells in the mouse brain, are methods which have been used to identify mechanisms. Describing these studies, in the rodent, we refer to bRG-like cells as those that are localized in basal regions, possessing a basal process, expressing bRG markers such as Pax6, Sox2, and pVim and capable of undergoing multiple rounds of division. Hence, listed in this section are the genes and signaling pathways which were shown to be involved in bRG/bRG-like cell generation (also resumed in Figure 2 and Tables 1, 2), with an update on the cellular mechanisms involved (Figure 3).

**TABLE 1:** List of genes involved in bRG generation.

**Gene/Protein**	**Function**	**Model**	**Manipulation**	**Effect(s) on progenitors**	**Cell mechanism described**	**Other phenotype(s) described**
ARHGAP11B	?	Mouse (IUE+mRNA microinjection)	Expression	IPs↗, basal mitoses↗	Cell fate change in aRG to produce more BPs, Proliferation↗	Thicker SVZ, Cortical folds, cortical expansion
		Ferret (IUE)	Expression	IPs↘, bRGs↗	Oblique/horizontal division in aRGs, Proliferation↗	Cortical expansion, upper-layer neurons↗
Aspm	MT associated protein	Ferret (transgenic line)	KO	bRGs↗	Early delamination of aRGs	Thinner VZ, microcephaly with conserved cortical layers
		Mouse (transgenic line)	KO	?	Mislocalized centrosomes and aberrant ventricular lining	–
Cdh1	Cell adhesion molecule	Ferret (IUE)	Expression of dominant-negative form	aRGs↘, bRGs↗	Cell adhesion↘, Oblique/horizontal division in aRGs	–
FGFR1, MEK, ERK, ETV4	RTK, MAPK, TF	Mouse (IUE)	Pathway activation (constitutively active form of FGFR1, Mek, Etv4)	bRGs↗	Proliferation↗	Upper-layer neurons↗
FGFRs	RTK	Ferret (IUE)	Pathway inhibition	bRGs↘	Proliferation↘ in bRGs	Loss of cortical folds
GPSM2 (LGN)	Regulator of mitotic apparatus	Mouse (IUE/transgenic line)	Expression of dominant-negative form	bRGs↗	Oblique/horizontal division in aRGs	–
Hif1α	TF	Mouse	Regulation of the pathway (Hyperoxia during pregnancy)	IPs↗, bRGs↗	?	OSVZ-like region
INSM1	TF	Mouse (IUE)	Overexpression	IPs↗ in the VZ, bRGs↗	Cell detachment from VZ, disruption of adherens junctions	–
Pax6	TF	Mouse (IUE/transgenic line)	Forced expression in BPs	IPs↘, bRGs↗	Oblique/horizontal division in aRGs, Proliferation↗	OSVZ-like region, thicker cortex, upper-layer neurons↗
		Mouse (transgenic line)	KO	bRGs↗	Cell adhesion↘, mostly non-cell autonomous	–
PDGFD/PDGFR signaling	PDGF receptor/ligand	Human brain slices	Pathway inhibition (treatment with PDGFRβ inhibitor)	IPs↘, bRGs↘	?	–
		Mouse (IUE+PDGF-DD injection)	Expression (constitutively active form of PDGFRβ)/Pathway activation	bRGs↗		
PTEN	Phosphatase	Human organoids	Pathway activation (CRISPR/Cas9 targeting of PTEN)	bRGs↗	?	Increased volume and cortical folds
		Mouse organoids		?		Increased volume but no cortical folds
SMARCC1 (Baf155)	Chromatin remodeling factor	Mouse (IUE/transgenic line)	KO	IPs↘ (ectopic IPs in IZ), bRGs↗	Oblique/horizontal division in aRGs, regulates Pax6, cell adhesion↘	-
SmoM2	Shh pathway activator	Mouse (transgenic line)	Pathway activation (constitutively active form of SmoM2)	IPs↗, bRGs↗	Proliferation↗	Cortical folds, brain expansion
TBC1D3	RABGAP	Mouse (IUE/transgenic line)	Expression	aRGs↗, IPs↗, bRGs↗	Cell adhesion↘, proliferation↗	Cortical folds
		Human brain slices (electroporation)	KD	bRGs↘	?	–
TMEM14B	Kinase?	Mouse (IUE)	Expression	IPs↗, bRGs↗	Oblique/horizontal division in aRGs, Proliferation↗	OSVZ-like region, cortical folds
Trnp1	TF	Mouse (IUE)	KD	aRGs↘, IPs↗, bRGs↗	Cell adhesion?	Cortical folds

*In this table are indicated the genes, their function, the model in which they were tested, the type of experiment performed, the effect on progenitor cells, the cellular mechanisms indicated in each study and other phenotypes associated with bRG regulation. IUE, in utero electroporation; aRGs↘, IPs↘, bRGs↘, diminution of the proportion of aRGs, IPs, and/or bRGs; aRGs↗, IPs↗, and bRGs↗, increase of the proportion of aRGs, IPs, and/or bRGs; Cell adhesion↘, diminution of cell adhesion (generally associated with loss or diminution of expression of adherens junction proteins or other cell adhesion molecules). Proliferation↗, increase in the proportion of mitotic and/or cycling cells; upper-layer neurons↗, increase in the proportion of upper-layer neurons in the cortical plate.*

**TABLE 2:** List of genetic tools used to promote or impair bRG generation.

**Gene/Protein**	**Animals**	**Constructs**	**References**
ARHGAP11B	–	pCMV6-AC-ARHGAP11B (Origene, SC324558)	Florio et al., 2015
		pCAGGS-ARHGAP11B	Florio et al., 2016
Aspm	Aspm KO ferrets	–	Johnson et al., 2018
	Aspm KO mice		
Baf155	Baf155 fl/fl mice X Emx1:CRE mice	pCIG2-Cre-ires-eGFP	Narayanan et al., 2018
Cdh1		pCAG-DN-Cdh1	Martínez-Martínez et al., 2016
FGFR1, MEK, ERK, ETV4	–	pCAG-MekDD-IRES-EGFP, pCAG-Etv4-IRES-EGFP, pCAG-Etv5-IRES-EGFP, pCAG-FGFR1K656E-IRES-EGFP	Heng et al., 2017
FGFRs	–	pCAG-sFGFR3 (DN)	Matsumoto et al., 2017
Hif1α	Hif1(α fl/fl mice X Nestin:Cre mice)	–	Wagenführ et al., 2015
INSM1	–	pCAG-Insm1-IRES-mCherry, pCas9-Plekha7	Tavano et al., 2018
LGN	–	pCAG-Floxp-FLAG-LGN-C	Shitamukai et al., 2011
Pax6	Tis21:CreERT2 mice	pCAGGS–LoxP-GAP43-GFP-LoxP-Pax6-IRES-nRFP	Wong et al., 2015
	Pax6fl/fl mice X Emx1:CRE mice	pCIG2-Cre-ires-eGFP	Narayanan et al., 2018
PDGFD/PDGFR signaling	–	pCAG-PDGFD-IRES-GFP	Lui et al., 2014
PTEN	–	gRNA for CRISPR/Cas9 mediated editing of human PTEN: 5′-aaacaaaaggagatatcaag-3′; FUW-DN-AKT and FUW-CA-AKT lentivirus constructs	Li et al., 2017
		gRNA for CRISPR/Cas9 mediated editing of mouse Pten: 5′-agatcgttagcagaaacaaaagg-3′	
SmoM2	Gt(ROSA)26Sortm1(Smo/EYFP)Amc X GFAP:Cre	–	Wang et al., 2016a
TBC1D3	Nestin:TBC1D3	pCS2-cMyC-TBC1D3, pCAGGS-TBC1D3-IRES-EGFP	Ju et al., 2016
		pSuper-siTBC1D3	
TMEM14B	–	pCMV-3 Falg-TMEM14B-EGFP; pCMV-3 Falg-IQGAP1-EGFP	Liu et al., 2017
Trnp1	–	pSuper.GFPNEO_shRNA	Stahl et al., 2013
		rv:DN-Trnp1	Martínez-Martínez et al., 2016

### FGF-MAPK Pathway

The MAPK pathway is involved in many processes such as proliferation, differentiation, migration and apoptosis. It is generally activated by soluble growth factors that interact with receptors, triggering a signaling cascade (e.g., Ras-Mek-Erk) that regulates many processes, including gene expression (Sun et al., 2015). It is therefore not surprising to find links between the MAPK pathway, cortical development and the generation of bRGs. Erk signaling has been shown to promote Bergmann glia generation and is important for cerebellar foliation (Li et al., 2014). As Bergmann glia are reminiscent of bRGs in several aspects (notably transcriptional signatures), Heng et al. (2017) investigated the role of the MAPK pathway in bRG generation. ERK signaling is strongly activated in humans as compared to the mouse. Interestingly, the authors showed that expression via *in utero* electroporation at E14 of constitutively active forms of Fgfr1 (a tyrosine kinase receptor known to activate the pathway), Mek (a MAP kinase) or Etv4 (a response gene of the MAPK pathway) all lead to increased generation of Hopx+/Pax6+/Sox2+ bRG-like cells in the mouse 2 days later, and these cells can produce neurons and astrocytes (Heng et al., 2017). bRGs produced with this method are akin to primate-like bRGs as they can efficiently proliferate (cells go through multiple rounds of divisions, producing clonal populations of neurons). Matsumoto et al. (2017) investigated whether FGF signaling was also involved in BP expansion in a gyrencephalic species, the ferret. They showed that several isoforms of FGFRs (FGFR1-3) are expressed in the developing cortex of the ferret at P0, when folding occurs. They also showed that *in utero* electroporation of a dominant-negative form of FGFR3 at E33 reduced cortical folding and reduced the proportion of IPs and bRGs in the oSVZ. This suggests that FGFR3 signaling promotes cortical folding and upper layer thickness by regulating BP (in particular bRG) production and proliferative properties. Overall, these results show that the FGF/MAPK/ETV axis is sufficient to promote the generation of bRG-like cells in the mouse and is important for bRG production and folding in the ferret.

### PDGFD Signaling

PDGF signaling was also shown to be important for bRG generation. As mentioned in section “Gene Expression Profile,” Lui et al. (2014) identified 18 genes specific to human radial glia through gene expression analyses from human embryonic brain slices. PDGFD, a secreted growth factor, was identified in that list. Inhibitors of PDGFRβ decrease the number of neural progenitors (SOX2+ and TBR2+) in human embryonic brain slices but not in the mouse. Conversely, intraventricular injection of PDGF-DD or expression of a constitutively active form of PDGFRβ via *in utero* electroporation increases the proportion of Sox2+ cells in basal zones in the mouse. PDGF signaling is suggested to involve the MAPK pathway (Papadopoulos and Lennartsson, 2018), thus PDGFD stimulation could lead to an increase of bRG-like cells in the mouse in an Erk-dependent fashion.

### PTEN/AKT

The PI3K/PTEN/AKT pathway is involved in cell proliferation and is well characterized in cancer (Carnero and Paramio, 2014). PI3K increases levels of PIP3 which activates AKT signaling. PTEN works in the opposite direction, as it is a PIP3 phosphatase. This pathway is involved in cortical development, especially described in pathologies such as microcephaly and macrocephaly when the pathway is impaired or activated, respectively (Jansen et al., 2015; Wang et al., 2016b). The team of Li et al. (2017) used CRISPR/Cas9 targeting of PTEN in human organoids to study its role in progenitors. Loss of PTEN induced an increase in volume, surface area and folding at the surface of the organoids clearly visible at 6 weeks and beyond, when control organoids appeared smooth. PTEN deletion in mouse organoids increased their size, but did not induce folds, suggesting that AKT signaling regulates human-specific mechanisms. At early stages (4–6 weeks), human control organoids showed many DCX+ cells, while mutant organoids predominantly displayed Nestin+ cells. Loss of PTEN increased the proportion of cycling progenitor cells including SOX2+ and HOPX+ bRG cells. PTEN mutant organoids also showed decreased cell cycle exit at 4 and 6 weeks, and a decreased proportion of DCX+/EDU+ cells. All these results suggest that neuronal differentiation was delayed, consistent with an increased proportion of cycling progenitors. However, this delay was normalized in the following weeks (by week 8) with many cells acquiring neuronal fate. The presence of early folds when neurons are rare could be explained by the forces generated by the amplification of neural progenitors and the changed radial glia architecture. PTEN rescue experiments showed organoids displaying smooth surfaces and normal levels of proliferation. To further confirm AKT signaling involvement in folding, the authors treated the organoids with different known inhibitors of AKT signaling or expressed a dominant-negative form of AKT, showing that in each case, PTEN mutant organoids appeared smooth when AKT signaling was inhibited. All these results point to the importance of the PI3K/PTEN/AKT signaling pathway being a regulator of bRG generation, proliferation and cortical folding.

### Hif1α

It has been suggested that neural progenitor cells are precisely regulated by oxygen (O_2_) levels (Pistollato et al., 2007), involving the transcription factor Hif1α (which is activated by hypoxia). Hif1α was removed in the mouse brain using a Nestin-Cre mouse line, with pregnant mice at E14 exposed to 10% (hypoxia), 21% (normoxia), or 75% (hyperoxia) (Wagenführ et al., 2015). Hypoxia reduced brain volume while hyperoxia lead to an increase in brain volume, accompanied with an increased number of Tbr2+ cells and Sox2+ bRG-like cells in a region beyond the SVZ forming a new neurogenic and proliferative niche (potentially oSVZ-like). Hyperoxia also increased the proportion of mitotic cells located in the IZ that were pVim+/Sox2+ and also pVim+/Sox2+/Tbr2+, but did not affect the proportion of mitoses in the VZ, suggesting that aRG proliferation was not altered by hyperoxia. In the IZ, hyperoxia, compared to normal O_2_ levels, doubles the proportion of mitotic cells in a Hif1α-dependent fashion. Finally, O_2_ tension was shown to regulate cortical thickness and layering including the correct positioning of migrating neurons. Hyperoxia increased cortical thickness with more Ctip2+ neurons (in deep layers). Hif1α is known to be regulated by the Akt/mTOR pathway in oncogenesis, leading to the regulation of gene programs involved in angiogenesis, metabolism and cell proliferation (El-Gazzar et al., 2014). Since AKT signaling promoted bRG generation in human organoids, it is possible that the AKT/mTOR/HIF1α signaling pathway could be regulated in such a way as to promote bRG genesis, especially in species in which bRGs are abundant.

### SHH Signaling

Shh signaling is involved in cell differentiation and the epithelial-mesenchymal transition (EMT) (Zhang et al., 2016; Chen et al., 2017). The delamination of neurons and progenitors (IPs and bRGs) could be compared to EMT since it involves delamination from the neuroepithelium and migration toward basal regions. Shh defects have been linked with microcephaly, in which the size of the brain and the cortex are reduced. Wang et al. (2016a) reported that transgenic mice expressing a constitutively active form SmoM2 of the Smoothened receptor display brain expansion and cortical folding at P7. These animals have increased proportions of Tbr2+ cells and bRG-like cells that are primate-like (possessing a basal and/or an apical process and performing multiple rounds of division). aRGs display more oblique divisions when the Shh pathway is activated. Conversely, Smo mutant mice display microcephaly, with a decreased number of IPs and less oblique divisions. Interestingly, SHH signaling is strongly active during human embryonic development (Wang et al., 2016a). Wang et al. hence investigated whether SHH signaling could also promote bRG enrichment in human cerebral organoids. Indeed, activation of the SHH pathway was associated with an increased proportion of oblique divisions in aRGs and an increased number of bRGs. A very recent study investigated the role of Gpr161, a repressor of the Shh pathway (Shimada et al., 2019) in cortical development in the mouse. Conditional loss of Gpr161 in progenitors (deletion under the control of the Nestin promoter) causes various pathological phenotypes such as hydrocephaly and periventricular heterotopias. However, consistent with the previous study, an increased proportion of BPs (Tbr2+ IPs and Pax6+ bRG-like cells) could be found in basal regions. The proportion of mitotic cells in the SVZ and IZ was also increased. These studies therefore suggest that SHH activation can induce the amplification of bRGs.

### Pax6

*Pax6* is a critical gene involved in many processes during development of the central nervous system (reviewed in Manuel et al., 2015). It codes for a radial glia-specific transcription factor that promotes the transition of NE cells to aRGs (Götz et al., 1998) and regulates proliferative and neurogenic properties of aRGs (Götz et al., 1998; Mo and Zecevic, 2008; Suter et al., 2009). In humans, PAX6 has a critical function as early as during neuroectoderm specification, as embryonic stem cells destined to become NEs express Pax6 before Sox2, which is not the case in the mouse (Zhang et al., 2010). Pax6 deficient mice display aRGs with abnormal glial fibers, and Pax6 regulates genetic programs in NE and aRGs to tightly control differentiation and neurogenesis in the forebrain, but also in eye development for which Pax6 has been show to play a major role in the mouse and in Drosophila (Xie et al., 2013; Baker et al., 2018). Pax6 is no longer expressed in aRG daughter cells that acquire either IP or neuronal cell fate in the mouse. In fact, Tis21 is a transcription factor that is expressed in a subpopulation of aRGs that will perform indirect or direct neurogenic divisions, while aRGs lacking Tis21 tend to perform self-amplifying divisions (Iacopetti et al., 2002). Wong et al. (2015) investigated the effect of sustained Pax6 expression in the daughter cells of Tis21-expressing aRGs, after generating a Tis21-CreER^T2^ transgenic mouse line. By performing *in utero* electroporation in tamoxifen-treated animals at E13.5 with a plasmid expressing Pax6, they were able to express the gene specifically in the progeny of Tis21+ neurogenic aRGs. This experimental approach led 24 h later to an increased proportion of Pax6+ cells in the VZ and SVZ along with an increased proportion of proliferative cells in the SVZ. This was accompanied by a decrease of Tbr2+ and an increase of Sox2+, Pax6+, and pVim+ cells that possessed a basal process in the SVZ, as well as more oblique divisions of aRGs. Conditional Pax6+ expression therefore appears to change cell fate for aRG progeny by promoting bRG-like cell generation. Furthermore, cell cycle re-entry was also doubled in SVZ cells, suggesting that Pax6 sustained expression in BPs gives them primate-like proliferation and self-renewal features, as they perform multiple rounds of division. Consistent with other studies, bRG-like cell enrichment in this model also led to a thicker CP with an increase in the proportion of Satb2+ upper-layer neurons, both in electroporated mice or transgenic animals expressing Pax6 under the control of the Tis21 promoter. Therefore, Pax6-sustained expression in aRG daughter cells leads to the enrichment of bRG-like cells in the mouse, which further emphasizes the importance of Pax6 in cortical development, and especially how it regulates expression of its target genes in the context of bRG amplification.

### mSWI/SNF Subunits BAF170 and BAF155

Chromatin remodeling is an important mechanism that allows cell and tissue-specific gene expression patterns during development, as transcription factors require accessible chromatin to bind to the regulatory sequences of the target genes they interact with (de la Serna et al., 2006). This allows for lineage-specific promoters and enhancers to act and modulate cell fate, which is paramount during development (Alver et al., 2017; Vierbuchen et al., 2017). The mSWI/SNF complex, also known as the BAF complex, utilizes ATP-hydrolysis for energy to mobilize nucleosomes, and is critical for regulating cell proliferation and differentiation (Son and Crabtree, 2014). The complex is composed of many interchangeable elements including core subunits SMARCC1 (BAF155) and SMARRCC2 (BAF170), and ATPases BRG1 and BRM. The composition of the complexes varies depending on time and cell type. Mutations of BAF complex components have been associated with cancer (Hodges et al., 2019) but also brain development impairments and intellectual disability (Machol et al., 2019).

The BAF complex and specifically certain subunits have been shown to be critical for cortical development, regulating the progenitor pools and differentiation (Tuoc et al., 2013a, b; Son and Crabtree, 2014). Brg1 was shown to be critical for the differentiation of NEs into aRGs (Matsumoto et al., 2016). Baf155 is expressed early in NEs and aRGs, is important for their proliferation, and is replaced by Baf170 at the start of neurogenesis in dividing aRGs (Tuoc et al., 2013b). Baf170 loss of function in progenitors in the mouse (crossing Baf170^fl/fl^ mice with Emx1-Cre mice to induce the deletion of the gene from E9.5 in neural progenitors) led to greater cortical size with an expansion of the pool of Tbr2+ cells in the SVZ, while Baf170 conditional overexpression led to the opposite phenotype with depletion of the progenitor pool and reduced cortical size (Tuoc et al., 2013a). In the absence of Baf170, the BAF complex includes more subunits of Baf155, which promotes Pax6-dependent gene modulation. Tuoc et al. showed that Baf170 regulates Pax6 dependent gene expression by recruiting the REST corepressor complex. Double conditional KOs for both Baf155 and Baf170 using Emx1-dependent Cre expression led to dramatically affected cortical size and thinner germinal zones (Narayanan et al., 2015). Loss of both subunits resulted in a breakdown of the BAF complexes, leading to an overall increase of repressive epigenetic marks (H3K27me2 and H3K27me3), with subsequent overall gene repression. Finally, Baf170 KO in the developing and adult hippocampus showed alteration of the position and identity of progenitors in the dentate gyrus, enhanced gliogenesis and repressed neurogenesis (Tuoc et al., 2017). This suggests that epigenetic markers, regulated partly by the BAF complex, play a crucial role during several processes of cortical development (Narayanan et al., 2015; Nguyen et al., 2016). In a follow-up study, a Baf155 conditional KO (obtained by crossing Baf155^fl/fl^ with Emx1-Cre mice) led to an overall decrease of Tbr2+ cells, but also among the remaining Tbr2+ cells, the presence of ectopic IPs in the IZ (Narayanan et al., 2018). The total proportion of Pax6+ cells was not affected, yet ectopic Pax6+/pVim+ cells that resembled bRG-like cells were observed in the IZ, suggesting a delamination of aRGs or bRG-like cell generation. This was accompanied by increased vertical mitoses in aRGs. No effects on cortical size and layers were observed in this model.

The Baf155/Baf170 balance hence appears to play a crucial role in the generation of both IPs and bRG-like cells. A conditional KO of Baf155, or Pax6 floxed mice electroporated with a Cre-expressing plasmid at E14.5 both promote the generation of bRG-like cells (with a stronger effect observed in a non-cell autonomous fashion). The authors deciphered this pathway by showing that Baf155 regulates bRG generation in a Pax6-dependent fashion by downregulating adherens junction-related genes, including genes coding for Cdc42 effector proteins 1 and 2 (Cep1 and 4, respectively). Knockdown of Cep4 via electroporation increased the proportion of IPs and bRG-like cells, mostly in a non-cell autonomous fashion. Finally, some genes enriched in human bRGs were shown to be upregulated in Baf155 KO animals, including Foxn4. Forced expression via *in utero* electroporation in the mouse yet again boosted Tbr2+ IPs and Pax6+ bRG-like cells in the mouse cortex. Finally, to further characterize how bRG production may be regulated by Baf155, Tuoc lab’s studied how RNA methylation might impact Baf155 mRNA stability (Zhao et al., 2016; Xie et al., 2019). They showed that RNA-binding motif protein 15 (RBM15) binds to and enriches m6a methylation, and in the mouse developing cortex, high levels of Rbm15 and low levels of Baf155 were found in the IZ and CP, and the converse situation in the VZ and SVZ, suggesting that methylation of Baf155 transcripts by Rbm15 may negatively regulate their half-life. Overexpression of Rbm15 via electroporation at E13.5 led to decreased levels of Baf155 in the VZ and to the presence of Pax6+/Sox2+ cells in basal regions in a non-cell autonomous fashion, consistent with Baf155 KO experiments mentioned above. Finally, the authors showed that Rbm15-dependent downregulation of Baf155 led to reduced Pax6-dependent activation of genes involved in adherens junctions and cell to cell interactions. Overall, these results suggest that there may be precise control of BAF155 mRNA through methylation, which in turn could modulate chromatin remodeling processes. Allowing certain transcription factors to promote or repress the expression of key genes could therefore be critical for progenitor function and generation of bRGs.

### INSM1

A major component of BP production appears to be delamination from the VZ, i.e., loss of attachment to the adherens junction belt. Tavano et al. (2018) studied the role of Insm1 [a transcription factor involved in the generation of BPs (Farkas et al., 2008)] in this process. Insm1 is present in a subset of VZ nuclei and the proportion of Insm+nuclei increases from E10.5 to E16.5 in the mouse, whereas expression in the SVZ is weak. Both in the mouse (E14.5) and the human developing cortex (between GW11 to 16), the majority of Insm+ nuclei in the VZ are also Tbr2+, suggesting these cells are newborn IPs. However, in humans, the proportion of INSM1+/TBR2+ cells decreases during corticogenesis, with a corresponding increase of INSM1+/HOPX+ cells, suggesting that INSM1 is also expressed in newborn human bRGs. This suggests that INSM1 could potentially be involved in the increased complexity of the BP pool in humans as compared to mice. Insm1 overexpression at E12.5 via *in utero* electroporation in the mouse increased the proportion of Tbr2+ cells in the VZ with no increase in the SVZ and IZ 1 day later. Insm1 overexpression increased abventricular centrosomes (correlated with increased detachment of cells), and daughter pair analyses after mRNA microinjection showed an increase of the proportion of cell couples with only one, or no cell retaining their apical contact to the ventricular surface. Of the detached cells, almost 50% displayed typical bRG morphology and almost all were Sox2+. Insm1 overexpression hence promoted aRG delamination, leading to bRG-like cell generation (40% of cells that delaminated after Insm1 forced expression retained radial glia morphology). FACS followed by RNA sequencing showed 640 genes whose expression was regulated by Insm1 forced expression, some of which are involved in cell adhesion and migration. Among the genes that regulate these functions and have an involvement at adherens junctions, the authors focused on *Plekha*7, which is expressed in the most apical region of the VZ and is downregulated after Insm1 overexpression. They showed that disruption of Plekha7 with a CRISPR/Cas9 approach phenocopies Insm1 overexpression with a delamination of progenitors, increasing the proportion of BPs, including cells with a basal process, further suggesting that loss of cell adhesion in the VZ promotes progenitor delamination, and bRG-like cell production.

### GPSM2 and Notch-Delta

As mentioned previously, mitotic spindle orientation in the VZ could also be a key factor in the generation and detachment of newly generated bRGs, to allow them to migrate and disperse in basal regions of the developing cortex. Mitotic spindle orientation has been described to be of critical importance for cell fate (di Pietro et al., 2016), including in the cortex (LaMonica et al., 2013; Matsuzaki and Shitamukai, 2015). With this in mind, the team of Shitamukai et al. (2011) studied the impact of the Notch-Delta pathways and the involvement of GPSM2 (mentioned as LGN in the study) on the control of mitotic cleavage orientation. They showed that expression of a constitutively inactive form of GPSM2 in the mouse embryo after *in utero* electroporation increased the number of oblique and vertical divisions in aRGs, producing more daughter cells inheriting the basal process and migrating to basal regions. Consistent with Wang et al. (2011), they also showed that oblique divisions occurred in normal mouse brains, albeit being rare events. Artificially generated cells (after GPSM2 inactivation) were Pax6+, and found to have a similar morphology to human bRGs. Overactivation of the Notch-Delta pathway via overexpression of the Notch intracellular domain (NICD) leads to an increased conservation of APs and maintenance of newly generated bRG-like cells (capacity of performing multiple rounds of division). Furthermore of general interest, in this study authors showed that after aRGs perform mitosis, daughter cells that did not inherit the basal process were as likely to become aRGs as the ones that inherited it, by regrowing a process toward the basal surface.

### ASPM

Abnormal Spindle Microtubule Assembly (ASPM) codes for a protein involved in normal mitotic spindle function in neural progenitors (Fujimori et al., 2014). It is the most common recessive microcephaly gene in human. Johnson et al. (2018) generated Aspm mutant ferrets by injecting genome editing constructs in ferret zygotes. The mutant animals display microcephaly that is reminiscent of the human pathology (reduction of brain size with preserved cortical layers). Homozygous mutants displayed a thinner VZ and thicker oSVZ at E35 (at the beginning of neurogenesis), with an increased proportion of displaced Pax6+, Sox2+/Ki67+ cells basally (in the oSVZ), as well as Tbr2+ cells. Many of the displaced Pax6 cells resembled bRGs (basal process, pVim+, pH3+, Ptprz1+, and Hopx+). RNA sequencing showed that aRGs, endogenous bRGs and displaced bRGs are transcriptionally indistinguishable in this ferret model. All these results suggest a possible early enrichment of bRGs at the expense of aRGs depleting the pool of progenitors, either by aRG detachment from the ventricular surface or by cell division in which one daughter cell becomes a bRG, and the other one either a neuron or an IP. This depletion of the progenitor pool could explain the microcephaly. Interestingly, knockout mice for Aspm showed an increased proportion of Tbr2+ IPs at the expense of aRGs, but did not in this case display ectopic Pax6+ or Sox2+ cells in basal regions (Johnson et al., 2018). This suggests that simply affecting mitotic spindle function is not sufficient to generate bRGs in an organism that naturally produces very few of them. Furthermore, ASPM interacts with the apical polarity complex, including PKCζ, and centrosome proteins. Indeed, Aspm -/- mice show mislocalized centrosomes and an aberrant ventricular surface, which could potentially explain why BPs are enriched in the ferret and the mouse (Johnson et al., 2018).

### Human and Primate Evolutionary Inventions

In gene expression studies, some genes that were shown to be specifically expressed in bRGs were present only in humans or higher primate cells. Among these genes, the roles of TBC1D3, TMEM14B, and ARHGAP11B in the generation of bRGs were investigated.

#### TBC1D3

The team of Ju et al. (2016) studied the role of hominoid-specific *TBC1D3*, a gene coding for a member of a RABGAP family. The interest of this gene is that it is present in multiple copies on human chromosome 17, present in one copy in the chimpanzee and absent in other species, making it a potential candidate gene that could contribute to the increased complexity of the human brain. Expressing TBC1D3 using *in utero* electroporation in the mouse embryonic brain at E13.5 increased the proportion of IPs and basal mitoses, including abundant bRG-like cells that were Pax6+ and Hopx+. Interestingly, expression of TBC1D3 in all progenitors, via electroporation or in transgenic mice expressing the gene under the control of the nestin promoter, induced the apparition of pseudo-folds on the surface of the brain in early postnatal mice. Expression of TBC1D3 influences the MAPK pathway, increasing levels of p-ERK, as well as downregulating Trnp1. *Trnp1* is a gene expressed in a subset of human and mouse aRGs that self-amplify and it regulates cell fate (Stahl et al., 2013). Stahl et al. (2013) overexpressed Trnp1 in the mouse using *in utero* electroporation (at E13) and showed an enrichment of Pax6+ APs at the expense of the generation of Tbr2+ BPs at E16, and conversely, knocking down the gene induced the amplification of Tbr2+ cells in the SVZ, and most importantly of cells carrying the hallmarks of typical human bRGs 3 days after electroporation. Cells were Pax6+, localized outside of the VZ, and possessed a basal process extending to the pial surface. These cells could divide, with one daughter cell retaining the basal process. Knocking down Trnp1 also led to the apparition of pseudo-circumvolutions on the surface of a subset of brains at post-natal day 2 (P2) and P5, similar to TBC1D3 overexpression. Trnp1 was shown to be a nuclear factor, and lowering its expression led to reduced gene expression levels of bHLH transcription factors, chromatin remodeling proteins, histone variants, as well as some anchoring and adhesion molecules such as Cdh6 and Cdh11, potentially suggesting that Trnp1 can promote cell retention in the VZ, which is consistent with aRG enrichment following Trnp1 overexpression. TRNP1 expression in human fetal cortex varies along the VZ, low expression levels correlate with zones of radial expansion (Martínez-Martínez et al., 2016). In the ferret brain, Martinez-Martinez showed temporal regulation of Trnp1 and Cdh1 expression allowing bRGs to be produced during an early time window (mentioned previously and in section “Gene Expression Profile”). The link between Trnp1 and the MAPK pathways remains unknown, but it is possible that TBC1D3 downregulates Trnp1 expression via Erk signaling (Ju et al., 2016).

Further related to cell adhesion in the VZ, Martínez-Martínez et al. (2016) reported ferret genes that were differentially expressed during bRG production (E34–E36). They found that Cdh1 was significantly lowered in expression just prior to peak bRG production from the VZ (lower at E34 than E30). *In utero* electroporation with a construct expressing a dominant-negative form of Cdh1 increased the proportion of Pax6+ cells in the SVZ at the expense of Pax6+ cells in the VZ, further suggesting that changing cell adhesion in the VZ and at the ventricular surface promotes bRG production. This also led to an increased proportion of oblique divisions in aRGs. Expressing Trnp1 in this paradigm prevented the apparition of these abundant bRGs, further suggesting the importance of maintaining low levels of Trnp1 to produce bRGs.

TBC1D3 also has an impact on cell adhesion since TBC1D3 overexpression in the mouse decreased N-cadherin expression in the VZ (Ju et al., 2016). N-cadherin levels are known to control aRG proliferative properties (Miyamoto et al., 2015). Linked to the increase of basal mitoses, co-expression of TBC1D3 with a construct expressing N-cadherin completely prevented the increase in abventricular divisions (and hence cells leaving the VZ), revealing the importance of TBC1D3 in influencing adhesion. Overall, TBC1D3 was shown to downregulate Trnp1 and N-cadherin, which led to bRG amplification and apparition of folds on the surface of the brain. These results suggest that TBC1D3 could be a key player in the complexification of the human brain, regulating critical processes of cortical development.

#### TMEM14B

Liu et al. (2017) performed RNA sequencing on human fetal brain samples, identifying bRG-specific genes, some not listed in previous studies, such as TMEM14B, KCNK10, DAG1, and HP1BP3. TMEM14B codes for a transmembrane protein present in primates. Its electroporation in the mouse at E13.5 cell autonomously induced the enrichment of Tbr2+ and Pax6+ BPs that showed basal processes, were localized in the SVZ and showed increased proliferation at E15.5. Mild cortical folding was observed, along with an increase in the proportion of Satb2+ cells, related to an increase in the proportion of upper-layer neurons. To express TMEM14B in all RGs, transgenic animals were generated that express TMEM14B under the control of the Nestin promoter using a conditional knock-in (CKI) approach. *In utero* electroporation and transgenic mice results were similar, showing at E15.5 an increased proportion of Tbr2+ cells, basally located Pax6+ and Pax6+/pVim+ cells in the SVZ and IZ (potentially forming an oSVZ-like region). There was an overall increase of neural progenitors with increased proliferation. Both *in utero* electroporation and CKI induced cortical folding, however the effect was as expected much stronger in the CKI model (with an increased number of cells in upper and lower neuronal layers). To understand how TMEM14B expression promotes BP amplification, bRG-like cell generation in the mouse and cortical folding, mass spectrometry was performed using transfected HEK cells to study the TMEM14B interactome, and the Ras-activating-like protein IQGAP1 was identified. *In utero* electroporation of a construct expressing IQGAP1 partially phenocopied TMEM14B expression. It was shown that TMEM14B promotes IQGAP1 phosphorylation and subsequent nuclear localization.

#### ARHGAP11B

In transcriptome analyses from fetal human and mouse neocortices to identify human-specific genes underlying bRG expansion, *ARHGAP11B* was revealed to be expressed both in human aRGs and bRGs, but not in neurons (Florio et al., 2015). The gene derives from partial duplication of *ARHGAP11A*, coding for a Rho GTPase, after divergence from the chimpanzee. However, ARHGAP11B does not have Rho GTPase activity. After electroporation at E13.5 of a construct expressing ARHGAP11B, mouse brains showed an increased proportion of Tbr2+ cells at E15.5, and daughter cell analyses after microinjection in aRGs showed that ARHGAP11B promoted cell detachment and symmetrical division to produce two Tbr2+ cells. Also, some electroporated brains showed cortical folding. ARHGAP11B-dependent BP enrichment requires a specific splice donor site in the *ARHGAP11B* gene which is absent in the ancestral gene (Florio et al., 2016). It allows the protein to have a particular C-ter domain thought to be essential for ARHGAP11B to promote BP and bRG production. ARHGAP11B was later expressed by *in utero* electroporation in the ferret embryo at E33, where bRGs are naturally abundant (Kalebic et al., 2018). The number of BPs was increased at P0, including cycling and mitotic cells in the SVZ (particularly in the oSVZ). The proportion of Sox2+ cells was increased but the proportion of Tbr2+ cells was decreased in the SVZ. Primate like Sox2+/Tbr2- bRGs were thus increased. Overall, by using BrdU and EdU injections, the study showed that ARHGAP11B expression in the ferret extended the neurogenic period as compared to control animals. Consequently, ARHGAP11B increased the proportion of upper layer neurons with an increased proportion of Satb2+ neurons (Kalebic et al., 2018). Overall, this data shows that ARHGAP11B expression promotes BP generation differentially between mouse and ferret, with increased Tbr2+ cells in the mouse, and increased bRGs in the ferret, with both species showing cortical expansion. This human-specific gene could therefore also be a good candidate to help explain bRG amplification during human cortical development.

## Cerebral Organoids – an Emerging Human *In Vitro* Model to Study bRGs

Recent advances in stem cell technologies enable the generation of human cerebral organoids derived from pluripotent stem cells (PSC). Today, several protocols to generate standardized and region-specific brain organoids are available representing different brain regions including dorsal and/or ventral telencephalon (reviewed in Marsoner et al., 2018). They all start by differentiating human PSCs as self-organizing 3-dimensional embryoid bodies. Most of the cortical region-specific organoids are generated under the influence of small molecule pathway modulators including SMAD signaling inhibitors to prevent mesoderm and endoderm differentiation, WNT signaling inhibitors to avoid posteriorization and SHH inhibitors to maintain dorsal identities (Kadoshima et al., 2013; Qian et al., 2016; Bagley et al., 2017). These organoids show similar 3D organization, histological layers, migration patterns as well the transcriptional profile of the early human developing brain (reviewed in Di Lullo and Kriegstein, 2017). First indications that bRGs are present in human cerebral organoids were made by the detection of SOX2+/PAX6+ proliferating cells located away from the ventricular-like zone (Kadoshima et al., 2013; Lancaster et al., 2013). These cells were described to exhibit a basal process extending toward the pia but to lack an apical process. In addition, the majority of these cells were shown to exhibit a horizontal cleavage angle. The molecular identity of bRGs could, however, first be reliably identified when molecular markers were established (Pollen et al., 2015). Comparative single cell transcriptome analyses of cerebral organoids with primary human brain tissue confirmed the presence of cells with bRG expression signatures in organoid cultures (Camp et al., 2015; Bershteyn et al., 2017). The structural positioning of bRGs within organoids were further investigated by Qian et al. (2016) who describe that organoids, similar to the developing human brain, exhibit an expanded SVZ-like zone, which is split by a thin gap into an iSVZ composed of TBR2+ IPs and an oSVZ. SOX2+ cells within the oSVZ were described to co-express the bRG markers HOPX, FAM107A, and PTPRZ1 and many of these cells were shown to actively divide. A further study investigating the characteristic mitotic behavior of bRGs in organoids by time-lapse confocal microscopy revealed that bRGs within the organoids display mitotic somal translocation (Bershteyn et al., 2017), the typical mitotic behavior for this cell population as mentioned in section “bRG Division: Mitotic Somal Translocation” (Lui et al., 2011), further supporting their cellular identity.

Although in human cerebral organoids, bRGs are proportionally fewer in number compared to respective human fetal tissue (Camp et al., 2015), they are potentially useful to identify and/or validate signaling pathways involved in the evolutionary expansion of this cell population. Watanabe et al. (2017) investigated the effect of the (LIFR)/STAT3 pathway on bRGs in cerebral organoids, this pathway being described to be expressed in bRGs of human and macaque, and to impact cell cycle progression (Pollen et al., 2015). They found that augmented stimulation of the (LIFR)/STAT3 pathway lead to a >3-fold increase in bRGs, thickening of the SVZ-like zone, and enhanced formation and separation of cortical layers thus supporting the importance of this pathway for bRG expansion (Watanabe et al., 2017). The impact of the SHH signaling pathway, which was described to be strongly active in human fetal neocortex, on bRGs was also tested in human cerebral organoids. However, even though this study reported a decrease in bRGs when applying a Smoothened (SMO) antagonist, adding a SMO agonist to the organoid cultures did not change bRG production. This differential efficacy of the SMO antagonist and agonist most likely reflects the already high levels of endogenous SHH signaling in the organoid cultures (Wang et al., 2016a) (described above in section “SHH Signaling”). The PTEN-AKT signaling pathway was also suggested to impact bRG proliferation in human, but not mouse, cerebral organoids (described in section “PTEN/AKT”) (Li et al., 2017). Further comparison of human cerebral organoids and human primary cells to chimpanzee organoids and primary macaque cells suggest that the PI3K-AKT-mTOR pathway is strongly active in human bRGs, possibly promoting bRG expansion (Pollen et al., 2019). In addition, the mTOR effector phosphorylated ribosomal protein S6 (pS6), which was reported to strongly immunolabel bRGs in the human developing brain (Nowakowski et al., 2017), was found to also strongly immunolabel bRGs in human organoids, while chimpanzee organoid- and primary macaque-derived bRGs showed only faint pS6 immunoreactivity (Pollen et al., 2019). Thus these studies not only provide candidate molecular mechanisms governing human-specific changes in bRG signaling pathways but also highlight that organoids can serve as a platform for systematically characterizing features of human bRGs during development, evolution and pathology (e.g., for the latter, see Bershteyn et al., 2017). It is, however, important to note that organoids have their limitations with respect to fully reproducing the *in vivo* situation of the human developing brain. Protocols need to be further developed to improve amongst others the anatomy, the interconnection of different brain regions, the maturation and cellular diversity. But even though the organoid technology is still at its infancy further developments will presumably allow researchers to investigate previously experimentally inaccessible processes of human brain development *in vitro*.

## bRgs and Pathology

A lot remains to be understood when it comes to the role of bRGs in normal cortical development and pathology. Genetic manipulation serves as a driving force to push either the mouse or the ferret neocortex to have more bRGs and more proliferation, serving as models to study normal human cortical development. But strikingly this is not always the case. For instance, concerning Aspm, early conversion of aRGs into bRGs in the ferret leads to a deleterious phenotype (Johnson et al., 2018), in which depletion of the pool of aRGs to promote bRG-like cell production led to microcephaly. However, mice mutated for Aspm (generated by gene-trap) which display microcephaly, show no obvious effect on mitotic spindle orientation and asymmetric vs. symmetric divisions in progenitors (Pulvers et al., 2010). This suggests either that truncated Aspm could exert some of its functions, or that there are species-specific mechanisms. Macrocephaly and microcephaly are suggested to be caused by progenitor malfunction (Desikan and Barkovich, 2016; Romero et al., 2018). Whether mutations leading to brain size defects in humans specifically affect bRGs remains unknown. Nevertheless, maintenance of aRGs, the core progenitors during cortical development, and correct timing and rate of production of bRGs, are critical to achieve the correct balance of all types of progenitors, and alteration of these processes could lead to these pathologies (Uzquiano et al., 2018). Also, our lab previously described that the brains of *HeCo* mice, mutated for Echinoderm Microtubule Associated Protein–like 1 (Eml1), display heterotopia (Kielar et al., 2014). Brains are characterized by clusters of cells (both early and late born neurons) which are retarded in the IZ during embryogenesis. Late-born neurons are finally trapped in these subcortical regions (the white matter) in adults. Human patients with mutations in EML1 also show ribbon-like heterotopias (Romero et al., 2018). Neuronal migration itself did not appear affected in these mice during corticogenesis, but abnormally located Pax6+/pVim+ progenitors were observed in the IZ and CP. This phenotype was also associated with an increase in the proportion of oblique divisions in aRGs. However abnormally located cells were found to not always be radially oriented suggesting basal detachment. Ectopically generated neurons from these abnormal basal Pax6+ progenitors are likely to contribute to the heterotopias in these mice. Shh activation in the mouse via the Gpr161 conditional KO also induced heterotopias that are accompanied with “bRG-like” cell amplification (Shimada et al., 2019). Furthermore, Pax6 loss of function in the *small eye* mouse mutant also displays basally located progenitors that carry RG characteristics, which is consistent with the results obtained in the Pax6 conditional mutants (Götz et al., 1998; Narayanan et al., 2018). Other mutations have also been shown to be associated with the presence of ectopically located progenitors (reviewed by Bizzotto and Francis, 2015) such as Arl13b and Marcks mouse mutants, potentially showing subtle differences from bRG-like cells. Alteration of bRG proliferation and function could however, also be involved in lissencephaly. While most of the previous studies mainly focused on neuronal migration impairment in these pathologies (Hirotsune et al., 1998; Tsai and Gleeson, 2005; Romero et al., 2018), little is known about the role of progenitors and especially bRGs (Yingling et al., 2008; Bershteyn et al., 2017). Due to their abundant presence in gyrencephalic brains, and the formation of folds on the surface of the mouse cortex when they are artificially enriched, they have been suggested to be crucial for the development of circumvolutions on the surface of the brain. Therefore, one can assume that they could be involved in pathologies in which gyri and sulci are not formed. However, it is also known that the presence of bRGs does not always strictly correlate with cortical folds, as the marmoset, which is a near-lissencephalic primate, possesses abundant bRGs and an oSVZ layer during corticogenesis (Hevner and Haydar, 2012; Kelava et al., 2012).

As mentioned in section “Gene Expression Profile,” the extracellular matrix could also play a role not only in the proliferative properties of bRGs but also in their generation. Indeed, each germinal zone expresses its specific pattern of these molecules (Fietz et al., 2012). Integrins are the main extracellular matrix receptors, mediating cell adhesion processes as well as initiating intracellular signaling cascades (Barczyk et al., 2010). Integrin α_v_β_3_ is expressed in RG basal processes at the level of varicosities, and disruption of α_v_β_3_ integrin in the ferret via echistatin or blocking antibody treatment leads to a decreased proportion of cycling Pax6+ cells in the SVZ (Fietz et al., 2012). However, earlier studies showed that disruption of β_1_ in the mouse, either via genetic KO in neural progenitors (Radakovits et al., 2009) or blocking antibody treatment (Loulier et al., 2009) leads to impaired cortical development. In these studies, proliferation of aRGs is affected, leading to reduced cortical size. The authors showed that β_1_ integrin loss of function increased the proportion of abventricular mitoses, due to the fact that the basal process detaches from the basal membrane and aRG cell bodies delaminate from the VZ. This delamination leads to abnormal basal processes, and eventually apoptosis. These results suggest that the extracellular matrix and its receptors are involved in the maintenance of the basal process, which plays potentially a crucial role in bRG generation, since basal process maintenance is a hallmark of these cells, as well as cell delamination from the VZ.

While enrichment of bRGs is generally associated with increased proliferation and is considered as a major factor in the process of cortical folding, the examples mentioned above showcase situations in which “bRG-like” cells are generated in deleterious conditions, or in models of human-related pathologies. It is important to revisit these pathologies with our current knowledge about bRGs as, for instance, the function of ectopic Pax6+ progenitors mentioned above and their contribution to the pathological phenotypes remain unclear in these contexts. Furthermore, their nature is also unknown, single-cell RNA sequencing could help us answer whether these cells have bRG identity (and characteristics) or if they are mislocalized and aberrant aRGs. Depletion of the AP pool, abnormal generation of neurons (proliferation and site of neurogenesis) could both affect normal corticogenesis. bRGs therefore represent a major subject for the scientific field and understanding how these cells are generated and function will help us understand better how the neocortex develops in humans, and therefore bring insights to help us fully understand human malformations of cortical development. This could potentially pave the way toward the development of new therapeutic strategies.

## Conclusion

This review offers a comprehensive view on bRGs, including mechanisms and genes currently described as being involved in their generation, their characteristics in terms of morphology and function as well as their role in the gyrification of the cortex. An intriguing phenomenon that is highlighted by mouse studies described in section “Molecular Mechanisms Associated With the Generation and Amplification of bRGs, Including bRG-Like Cells in the Rodent” is that enriched bRG-like cells behave like human cells when considering proliferation and progeny. This suggests that not only do these genes promote the cell machinery that is required for aRGs to massively generate bRGs, they also could activate a transcriptional program in newly generated bRG-like cells that is potentially distinct from mouse resident bRG-like cells.

Considering all the information mentioned above, another interesting feature of the studies that we highlighted is that each of the genes that were manipulated are individually sufficient to promote bRG-like cell generation when their expression and/or function are modified. For most of them, an expression level modification is sufficient to generate primate like bRG-like cells in the mouse by affecting different cellular mechanisms (Figure 3A and Table 1). Overall, the genes identified impact various signaling pathways and belong to gene families that possess different functions, however these all converge to promote bRG-like cells (Figure 2). This suggesting that either there exist multiple distinct signaling pathways and cellular mechanisms which are responsible for bRG generation from aRGs independent from one another (this could be the case impacting either spindle orientation or cell adhesion changes), or that all these genes are part of an all-encompassing pathway in which alteration of one actor impacts part or all the other actors in the network. It currently remains a challenge to have a global understanding of how these pathways interact together and regulate each other (Figure 2), and thus it is impossible today to have a definitive answer to this question.

However, even though bRGs (or bRG-like cells) are amplified in all cases mentioned, the studies describe different consequences that are associated with bRG enrichment, such as depletion of certain pools of progenitors or folding on the surface of the mouse brain, depending on the gene involved and the model used (Figure 3B). This may suggest that not all the bRG-enriching methods are equal, potentially contradicting the all-encompassing hypothesis. Considering the current trend of this field of scientific research, we can expect in the near future more bRG generation genes to be identified and tested, which will further reveal the potential interrelations between these signaling pathways.

## Author Contributions

All authors listed have made a substantial, direct and intellectual contribution to the work, and approved it for publication. JL wrote the human organoid section. MP, RB, and FF wrote the rest of the manuscript.

## Conflict of Interest Statement

The authors declare that the research was conducted in the absence of any commercial or financial relationships that could be construed as a potential conflict of interest.
