# Corrigendum: Genes and Mechanisms Involved in the Generation and Amplification of Basal Radial Glial Cells

**DOI:** 10.3389/fncel.2019.00462

**Published:** 2019-10-21

**Authors:** Maxime Penisson, Julia Ladewig, Richard Belvindrah, Fiona Francis

**Affiliations:** ^1^Inserm, Institut du Fer à Moulin, Sorbonne Université, Paris, France; ^2^Inserm UMR-S 1270, Paris, France; ^3^Institut du Fer à Moulin, Paris, France; ^4^Central Institute of Mental Health, Medical Faculty Mannheim, Heidelberg University, Mannheim, Germany; ^5^Hector Institute for Translational Brain Research (gGmbH), Mannheim, Germany; ^6^German Cancer Research Center, Heidelberg, Germany

**Keywords:** cortical development, neural progenitor cells, basal radial glia, cell division, spindle orientation, adhesion, signaling pathways

In the original article, the citation “(Kalebic et al., 2018)” was not cited in the article. The citation has now been re-inserted in the section ***Molecular Mechanisms Associated With the Generation and Amplification of bRGs, Including bRG-Like Cells in the Rodent***, subsection ***Human and Primate Evolutionary Inventions***, paragraph six ***ARHGAP11B***. The new text is as follows:

“In transcriptome analyses from fetal human and mouse neocortices to identify human-specific genes underlying bRG expansion, *ARHGAP11B* was revealed to be expressed both in human aRGs and bRGs, but not in neurons (Florio et al., 2015). The gene derives from partial duplication of *ARHGAP11A*, coding for a Rho GTPase, after divergence from the chimpanzee. However, ARHGAP11B does not have Rho GTPase activity. After electroporation at E13.5 of a construct expressing ARHGAP11B, mouse brains showed an increased proportion of Tbr2+ cells at E15.5, and daughter cell analyses after microinjection in aRGs showed that ARHGAP11B promoted cell detachment and symmetrical division to produce two Tbr2+ cells. Also, some electroporated brains showed cortical folding. ARHGAP11B-dependent BP enrichment requires a specific splice donor site in the *ARHGAP11B* gene which is absent in the ancestral gene (Florio et al., 2016). It allows the protein to have a particular C-ter domain thought to be essential for ARHGAP11B to promote BP and bRG production. ARHGAP11B was later expressed by *in utero* electroporation in the ferret embryo at E33, where bRGs are naturally abundant (Kalebic et al., 2018). The number of BPs was increased at P0, including cycling and mitotic cells in the SVZ (particularly in the oSVZ). The proportion of Sox2+ cells was increased but the proportion of Tbr2+ cells was decreased in the SVZ. Primate like Sox2+/Tbr2- bRGs were thus increased. Overall, by using BrdU and EdU injections, the study showed that ARHGAP11B expression in the ferret extended the neurogenic period as compared to control animals. Consequently, ARHGAP11B increased the proportion of upper layer neurons with an increased proportion of Satb2+ neurons (Kalebic et al., 2018). Overall, this data shows that ARHGAP11B expression promotes BP generation differentially between mouse and ferret, with increased Tbr2+ cells in the mouse, and increased bRGs in the ferret, with both species showing cortical expansion. This human-specific gene could therefore also be a good candidate to help explain bRG amplification during human cortical development.”

The reference list has also been updated accordingly.

The authors apologize for this error and state that this does not change the scientific conclusions of the article in any way. The original article has been updated.
